# Age, education and gender effects on neuropsychological functions in healthy Indian older adults

**DOI:** 10.1590/S1980-57642014DN82000010

**Published:** 2014

**Authors:** Ravikesh Tripathi, Keshav Kumar, Srikala Bharath, P. Marimuthu, Mathew Varghese

**Affiliations:** 1 Consultant Clinical Psychologist, Narayana Health, MS Medical Centre, Bangalore-99; 2 Additional Professor of Clinical Psychology, NIMHANS, Bangalore-560029; 3 Professor of Psychiatry, NIMHANS, Bangalore; 4 Additional Professor of Biostatistics, NIMHANS, Bangalore; 5 Professor of Psychiatry, NIMHANS, Bangalore

**Keywords:** neuropsychological tests, older adults, literacy and cognition

## Abstract

It is essential to use culturally appropriate, sensitive and specific tests that reflect true cognitive performance. However, several factors including age, education and gender can influence neuropsychological test performance.

**Objective:**

To examine the effects of age, education and gender on neuropsychological function in older adults using measures of global cognitive screening, attention, working memory, executive functions, memory, construction, language and parietal focal signs.

**Methods:**

This is a cross sectional normative study of 180 community-dwelling normal older adults. All participants were screened with the Hindi Mental Status Examination (HMSE), Everyday Activities Scale for India (EASI), Edinburgh handedness inventory (EDI) and MINI Screen, and followed by a detailed neuropsychological assessment.

**Results:**

Stepwise regression analysis revealed that education was associated with better performance on all the neuropsychological tests. Females performed significantly better on measures of memory. Further, most of the illiterate subjects, including low educated participants, refused to cooperate on measures of executive functioning.

**Conclusion:**

Education was found to be the strongest determinant of neuropsychological test performance followed by age and gender. Our study demonstrates that Indian healthy normal older adults with low education perform poorly on measures of planning and working memory. Traditional measures of planning and working memory should be avoided or used cautiously in the presence of low education. There is an urgent need to develop tasks for measuring executive functions, especially in low educated Indian older adults.

## Abstract (pt)

É essencial o uso de testes específicos, sensíveis e culturalmente apropriados que reflitam o verdadeiro desempenho cognitivo. Todavia, vários fatores como idade, educação e gênero podem influenciar o desempenho neuropsicológico em testes.

**Objetivo:**

Examinar os efeitos de idade, educação e gênero na função neuropsicológica em adultos idosos usando medidas de rastreio cognitivo global, atenção, memória operacional, funções executivas, memória, construção, linguagem e sinais parietais focais.

**Métodos:**

Este é um estudo normativo de corte transversal de 180 idosos vivendo em comunidade. Todos os paticipantes foram rastreados com o Exame do Estado Mental Hindu (HMSE), Escala de Atividades de Vida Diária para Índia (EASI), Inventário de Dominância Manual de Edinburgh (EDI) e MINI Screen seguidos por uma detalhada avaliação neuropsicológica.

**Resultados:**

A análise de regressão *stepwise* revelou que a educação esteve associada ao melhor desempenho cognitivo em cada teste neuropsicológico. As mulheres tiveram desempenho significativamente melhor em medidas de memória. Além disso, a maioria dos analfabetos incluindo idosos com baixo nível educacional recusou-se a participar em medidas de função executiva.

**Conclusão:**

Educação foi o mais forte determinante do desempenho em testes neuropsicológicos seguido por idade e gênero. Nosso esrudo demonstra que idosos indianos saudáveis com baixo nível educacional tem desempenho pobre em medidas de planejamento e memória de trabalho. Medidas tradicionais de planejamento e memória de trabalho devem ser evitadas ou usadas cautelosamente na presença de baixa escolaridade. Há necessidade urgente do desenvolvimento de tarefas de funções executivas especialmente para idosos indianos com baixo nível educacional.

## INTRODUCTION

Neuropsychological assessment plays a crucial role in identifying cognitive impairment associated with aging and dementia. However, cross-cultural neuropsychology as an emerging field of neuropsychology focuses more on cultural experience that could lead to performance differences unrelated to brain functioning.^1^ Therefore, culturally-appropriate, sensitive and specific tests have been recommended to reflect true cognitive performance, particularly when assessing the elderly population.^2-5^ Recently, there has been growing concern to develop culturally-specific norms for Asian populations, including India.^1-4^

The elderly populations from non-Western countries have rarely been exposed to testing situations and often lack test-taking attitude, which can result in lack of cooperation as well as poor test performance.^1-5^ In addition to familiarity with testing situations, socio-demographic correlates such as education, gender and age are also known to affect cognitive test performance. Conversely, literacy and educational level have been associated with better performance on neuropsychological tests.^6,7^ Participants with higher education performed better than participants with lower education on various cognitive domains including memory, problem solving, working memory, and visuo-constructional ability.^8-11^ Learning to read reinforces and modifies several fundamental abilities including perception, reasoning, memory, phonological awareness and visuo-constructional skills.^6^ With regard to gender, it has been observed that females perform better on verbal tasks while males outperform females on visuo-spatial tasks.^12-14^ It has been observed that while age-related cognitive decline is more pronounced in speed of processing, working memory and long-term memory,^14,15^ implicit memory and knowledge storage are relatively resistant to cognitive aging.^16,17^

The Indian population is unique in terms of wide variations in language, literacy and culture. Most of the older adults in India are unfamiliar with testing situations, a factor that could affect test performance. In India, attempts have been made to develop global cognitive screening and neuropsychological test batteries for older adults.^18-21^ However there is a paucity of relevant studies on socio-demographic correlates of neuropsychological functions. The purpose of the present study was to examine the role of age, education and gender on neuropsychological performance in Indian older adults. Further, we were interested in documenting the suitability of selected neuropsychological tests for use with Indian older adults. The present study is part of a larger ongoing study aimed at developing culturally-appropriate neuropsychological measures for Indian older adults.

## METHODS

The study sample consisted of 180 community-dwelling normal older adult, living independently in terms of their daily activities. All the participants were selected from the community or other support groups for the elderly, and agreed to participate in the present study. The study was approved by the local ethics committee (National Institute of Mental Health and Neuro Sciences). Written informed consent was obtained from each participant before initiating the study. All participants were screened with the Edinburgh handedness inventory, Hindi Mental Status Examination (HMSE), Everyday Activities Scale for India (EASI) and Modified MINI Screen and followed by a detailed neuropsychological assessment. Participants were excluded if they had history of neurological, neurosurgical or psychiatric illness; difficulty in activities of daily living (EASI score ≥1) or a HMSE score ≤19 for participant with education (up to 5 years) and <25 for 6 years or more of education.

**Edinburgh handedness inventory (Old Field, 1971).** The Edinburgh handedness inventory^22^ was used to determine handedness. This inventory adopts a brief and simple method of assessing handedness on a quantitative scale for use in neurological and other clinical and experimental work.

**Hindi mental-status examination (HMSE).** The Hindi mental-status examination (HMSE) is a modified version of the MMSE validated for the Indian population.^23^ In this study, the HMSE was used to screen participants with cognitive impairment (≤19 for 0 to 5 years of schooling; <25 for 6 years or higher).

**Everyday Abilities Scale for India (EASI).** This comprises a 12-item brief measure of activities of daily living, with norms, and is appropriate for use in evaluating dementia (together with other tests) among elderly in India.^24^ This scale was used to detect difficulty in activities of daily living.

**Modified MINI Screen.** The Modified Mini Screen is a set of 22 items derived from a structured psychiatric interview.^25^ This screening tool was used to screen individuals with major mental health disorders.

**Neuropsychological assessment.** A comprehensive and standardized neuropsychological battery (Table 1) was administered to all the participants.^19^ The reliability of each test included in the battery was established using the test-retest method and was found to be moderate to high (0.58-0.94) indicating satisfactory temporal stability of scores.^19^ The entire battery was found to be sensitive in differentiating normal controls from AD patients.^20^ The tests included in the battery are described here briefly. Episodic memory was assessed with a word list assessing immediate and delayed recall. Attention was assessed by a span task and picture cancellation task. Constructional ability was assessed with the stick construction test. Executive functions were assessed with the digit span, Corsi block-tapping test, Tower of Hanoi, fluency and Go/No-Go task. Language abilities were assessed by the picture naming test and semantic verbal fluency test. The neuropsychological battery takes 45 minutes to administer. All participant were administered the battery individually in the same order (Table 1).

**Table 1:** Neuropsychological tests used in this study.

	Test (s)	Description	Function (s)	Score min-max
1.	Word list^19,20^	Word List consists of ten semantically and phonetically unrelated words; three learning trials followed by delayed recall (after 20 minutes) and recognition.	Verbal memory functions; verbal learning and delayed recall; recognition.	Learning 0-30Recall 0-10Recognition 0-10
2.	Stick Construction Test^19,20^	Stick construction is a non-graphomotor task. The test consists of 5 constructions (stick designs), followed by immediate and delayed recall.	Constructional ability and visual learning and memory.	Construction-0-24Recall 0-24
3.	Digit span^29,20^	This is a test of attention/short term memory. It consists of 6 items for each in forward and reverse conditions.	Attention and working memory (Verbal).	Forward 3-8Reverse 2-7
4.	Corsi block-tapping test^29,20^	This test is a widely used paradigm to assess short-term and working memory using a nonverbal analog of the Digit Span procedure.	Attention and working memory (Visuo-spatial).	Forward 3-8Reverse 2-7
5.	Tower of Hanoi Test^29,20^	The tower of Hanoi is a widely used task to assess planning ability. In our study we have used items up to four disks.	Planning, Problem solving, response inhibition.	2 disk total move ≥3disk total move ≥7disk total move ≥15
6.	Category fluency^29,20^	The subject is required to produce as many names for a given category within a limited period of time. Three categories were used (Fruits, Vegetables and Animals) in the present study.	Language; executive function; semantic memory,	5-25
7.	Go/No-Go^29,20^	Go/No-Go test is an opposite responding task that requires suppressing a response to salient stimulus.	Response Inhibition.	0-10
8.	Parietal focal sign^19,20^	Consists of items for testing apraxia (ideomotor, ideational and construction), agnosia, acalculia and left-right disorientation.	Apraxia, Agnosia, Acalculia and Left-right disorientation	0-10
9.	Picture naming test^19,20^	Picture naming test consists of 24 line drawing pictures belonging to different categories such as animals, fruits, vegetables and objects relevant to daily life.	Language; semantic memory.	0-24

The Statistical Package for the Social Sciences (SPSS 12.0) was used to analyse the data obtained. Descriptive statistics by years of education, gender and age were reported. Stepwise multiple regression analysis was used to examine the contribution of age, gender and years of education on neuropsychological test performance.

## RESULTS

A total of 180 participants in the age-range of 55 to 64 years completed the assessment. Mean age of the participants was 59 years (SD=3.34) and mean education was 12 years (SD=5.60). All the participants were right handed. There were 18 illiterate participants (11 female) and 10 participants with between 1 and 5 years of education (Table 2). The descriptive statistics of the sample, stratified by Years of education, gender and age, are given in Table 3.

**Table 2:** Statistics of participants by years of education, age and gender.

Age	Years of education											
	Illiterate			1-5			6-12			≥13		Total
	Male	Female		Male	Female		Male	Female		Male	Female	
55-59	3	5		3	2		20	13		38	15	99
60-64	4	6		5	0		13	8		33	12	81
Total	7	11		8	2		33	21		71	27	180

**Table 3:** Means (M) and standard deviations (SD) stratified by years of education, age and gender

Variables	Years of education								
	Age in years	0-5 years			6-12 years			>12 years	
		Male	Female		Male	Female		Male	Female
Word list Learning Total	55-59	18.17±3.76	17.43±3.82		20.45±3.41	22.31±3.71		21.16±2.85	22.00±2.42
	60-64	16.22±3.03	16.67±3.14		18.08±3.89	21.50±1.41		20.85±2.45	22.00±3.43
Word list Delayed Recall	55-59	5.83±2.56	6.29±1.11		6.75±1.21	7.08±1.38		6.68±1.21	7.53±1.06
	60-64	5.11±1.76	6.17±1.17		6.0±1.35	7.50±0.53		6.58±1.29	7.42±1.62
Word list Recognition	55-59	8.83±0.98	9.57±0.53		9.90±0.31	9.78±0.60		9.70±0.66	9.93±0.26
	60-64	8.55±1.01	9.67±0.51		9.08±1.44	9.38±1.06		9.36±1.63	9.83±0.38
Fluency for Animals	55-59	13.50±4.32	15.14±2.73		13.85±4.18	15.73±3.13		15.24±3.78	15.14±3.30
	60-64	11.00±4.50	12.50±3.99		14.46±1.89	12.75±2.76		15.27±3.33	15.66±4.36
Digit Span (Forward)	55-59	4.00±0.89	4.14±0.90		5.35±1.60	5.38±0.96		6.13±0.91	5.60±0.99
	60-64	3.78±0.83	4.33±0.52		5.38±0.96	4.88±0.83		6.39±0.93	5.75±0.87
Digit Span (Reversed)	55-59	2.00±1.67	1.71±1.70		3.75±1.58	3.53±0.77		4.92±0.94	4.53±0.99
	60-64	1.89±1.17	1.50±1.64		3.77±0.72	3.50±0.76		4.76±1.03	4.92±1.08
Spatial Span (Forward)	55-59	5.17±1.17	4.29±1.25		5.20±1.47	5.77±0.73		6.08±0.75	5.60±1.06
	60-64	4.44±0.73	4.33±1.21		5.42±0.79	4.88±0.83		5.84±0.72	5.50±0.67
Spatial Span (Reversed)	55-59	2.67±1.97	2.57±0.53		4.15±1.50	4.54±1.13		5.05±1.06	5.00±1.07
	60-64	2.44±1.74	2.00±1.55		4.00±1.13	3.50±1.20		5.13±1.24	4.50±1.00
Stick Construction (Copy)	55-59	23.83±0.41	22.29±2.75		23.95±0.22	23.92±0.28		24.00±0.00	24.00±0.00
	60-64	22.56±3.24	20.17±6.74		24.00±0.00	23.88±0.35		24.00±0.00	24.00±0.00
Stick Construction (IR)	55-59	22.67±2.34	22.14±2.34		23.30±1.60	23.46±1.39		23.95±0.23	23.60±0.74
	60-64	19.44±6.44	18.83±9.52		22.38±2.26	22.38±2.56		23.94±0.24	23.58±1.16
Stick Construction (DR)	55-59	11.33±4.27	12.07±6.25		14.15±5.31	14.46±4.71		16.04±4.61	16.10±4.32
	60-64	9.67±5.77	8.83±7.08		15.27±3.83	13.75±3.96		16.77±4.16	16.67±5.04
Attention Test (TT)	55-59	240.8±63.98	247.4±63.98		176.27±29.80	157.08±50.70		156.37±30.86	163.31±31.60
	60-64	241.4±48.03	280 ±60		195.18±55.51	173.13±27.92		156.32±26.31	167.73±53.38
TOH Two disk Total Move	55-59	4.00±2.24	3.00±0		3.00±0.00	3.00±0.00		3.00±0.00	3.00±0.00
	60-64	3.01±0.33	3.02±0.45		3.00±0.00	3.00±0.00		3.03±0.35	3.00±0.00
TOH Three disk Total Move	55-59				8.77±1.36	14.89±6.27		11.83±6.18	13.00±5.75
	60-64				11.45±4.99	10.14±2.73		11.03±5.03	10.60±3.57
TOH Four disk Total Move	55-59				30.72±9.94	33.25±13.69		30.37±13.26	26.07±9.95
	60-64				23.50±6.02	27.00±10.17		29.23±1097	25.00±10.22

Stepwise regression was used to examine the effect of each independent variable (age, education and gender) on neuropsychological test variables. Table 4 shows the contribution of age, education and gender on neuropsychological tests. Education was found to be a strong contributor with a significant effect on all variables, accounting for 3%-48% of variance on each task. Gender was noted to be significantly associated with better performance on 2 tests, accounting for 4%-5% of variance on each task. Age was significantly associated with better performance on 3 tests, accounting for 2%-17% of variance on each task. The table indicates that the role of education is important in explaining the variability in test performance, followed by age and gender. No variability was detected between the groups on measures of agnosia, apraxia, naming, Go/No-Go and left right disorientation. Hence, these variables are not shown in the table.

**Table 4:** Contribution of age, education and gender on neuropsychological test performance

Dependent variables		Independent variables	B	T	P	R²	Proportion of attribution
1.	Verbal learning	Education	0.62	10.16	<0.001	0.40	0.34
		Age	-0.14	-0.24	0.015		0.02
		Gender	0.19	3.12	0.002		0.04
2.	Verbal memory	Education	0.62	10.00	<0.001	0.39	0.34
		Gender	0.23	3.71	<0.001		0.05
3.	Digit span (forward)	Education	0.59	9.82	<0.001	0.35	0.35
4.	Digit span (reversed)	Education	0.69	12.73	<0.001	0.48	0.48
5.	Spatial span (forward)	Education	0.49	7.42	<0.001	0.24	0.24
6.	Spatial span (reversed)	Education	0.60	10.03	<0.001	0.36	0.36
7.	Animals fluency	Education	0.28	3.88	<0.001	0.08	0.08
8.	Go/No-Go	Age	0.16	2.0	0.04	0.02	0.02
9.	Stick construction (copy)	Education	0.35	5.05	<0.001	0.13	0.13
10.	Stick construction (IR)	Age	-0.18	-2.75	<0.001	0.21	0.17
		Education	0.42	6.26	<0.001		0.04
11.	Stick construction (DR)	Education	0.42	6.16	<0.001	0.18	0.18

## DISCUSSION

Older adults from non-Western countries such as in India, often lack exposure to testing situations and test-taking attitude,^21^ consequently performing poorly on adapted tests.^3,4^ Several researchers have strongly recommended developing culturally appropriate, sensitive and specific tests, especially for assessing elderly populations.^26,27^ Recently, attempts have been made to develop culturally-appropriate tools for use in Indian older adults.^18-21^ However, the impact of demographic variables on neuropsychological test performance has not been adequately explored. The main purpose of the present study was to examine the relative contribution of age, education and gender on neuropsychological functions.

The results of the present study indicated that education was associated with better performance on all the neuropsychological tests. Increasing years of education was associated with higher scores on neuropsychological tests. The effects of education on neuropsychological test performance have been demonstrated by a number of studies.^6-12^

In our study, healthy older adults with lower educational levels performed poorly on the Tower of Hanoi and working memory tests, particularly on the backward conditions of the digit span as well as the Corsi block-tapping test. Surprisingly, our healthy normals with lower education (0 to 5 years) often refused to cooperate on the Tower of Hanoi, Digit span and Corsi block-tapping test (Reversed). Those who did cooperate were unable to move beyond the first or initial level of planning (TOH) and working memory (Reversed condition of span test), suggesting a possible ceiling effect. Several of the participants indicated that they were unexposed to mental operations, such as the reversing information or planning operations required for the TOH, in their everyday life. Further, they needed repeated prompts and instruction to complete the test. Several participants found the testing situation to be a learning opportunity to refine their skill during the assessment. This also implied that, given the opportunity, there was a possibility for improving their performance. Therefore, poor performance did not necessarily indicate disturbances in brain functions. This highlights the need for extreme caution while interpreting test findings.

There are several factors which could possibly explain the effects of education on neuropsychological tests. The low scores on neuropsychological tests obtained by low educated participants may be due to differences in learning opportunities, lack of exposure to psychological testing situations or because test items may be inferred as less meaningful and absurd, resulting in an inability to relate to the test.^6^ Formal schooling is known to improve test-taking attitude and facilitate the ability to learn and remember in a stepwise manner^7^ which in turn could contribute to better performance on neuropsychological tests. Higher education is associated with reduced risk of developing dementia and risk of memory decline in normal aging.^28^

Further, our study revealed that gender was a significant predictor for verbal learning and memory. In the present study, females scored higher than males on memory tasks. Female superiority on verbal memory is in line with other studies.^12,13^ With regard to age, we noted that age was a significant predictor of performance for several tests. This is consistent with previous studies that have indicated significant differences across age groups or significant correlations with age.^10,12^ One possible explanation as to why age had no effect on the performance of some neuropsychological tests in our study could possibly be due to the fact that our sample was relatively younger and homogenous.

This is one of few Indian studies exploring the effects of age, education and gender on cognitive functions and provides preliminary observations on the utility of selected measures for use in Indian older adults. The present study indicated that education is a crucial factor (other than gender and age) affecting performance in the Indian cohort. In our study, we employed standardized measures of attention, working memory, executive functions, memory, construction, language and parietal focal signs and noted that most of these selected measures were found to be appropriate and suitable for older adults. However, we observed that the Tower of Hanoi, Digit span and Corsi block-tapping test may not be useful for low educated older adults. Therefore, traditional measures of planning and working memory should be avoided or used cautiously in the presence of low education.

Our study is not devoid of limitations. The study involved primarily young older adults and hence its findings cannot be generalized to other age groups. Another important shortcoming of the present study was that we did not group illiterate participants in a separate category to subjects with low education (1-5 years of education). However, in our preliminary analysis no difference between these two groups was found. Hence, these two groups were merged into one category. Future studies are required to examine the impact of literacy levels and quality of education on neuropsychological functions. It would have been ideal to include an equal number of participants of both genders. Though attempts were made to recruit equal numbers, this was not possible due to the high rate of refusal to participate among potential female participants, particularly from low educated backgrounds. The refusal to participate could be attributed to lack of exposure to test situations or a lack of knowledge about research and its implications. In addition, voluntary female participation in some activities in rural India may be considered socially unacceptable whereas interacting with male strangers may be considered socially inappropriate.

In conclusion, education was found to be a strong contributor affecting neuropsychological performance, followed by age and gender. Measures of executive functioning should be used cautiously with low educated participants. Poor performance on some of the neuropsychological tests may not necessarily imply brain dysfunction, but could be attributed to culture and related experience. Therefore, neuropsychological test findings should be interpreted with caution. It is imperative to examine the suitability of adapted Western tests when testing cognitive function in the Indian population.
